# Exploiting RAS Nucleotide Cycling as a Strategy for Drugging RAS-Driven Cancers

**DOI:** 10.3390/ijms21010141

**Published:** 2019-12-24

**Authors:** Tyler E. Mattox, Xi Chen, Yulia Y. Maxuitenko, Adam B. Keeton, Gary A. Piazza

**Affiliations:** 1 Drug Discovery Research Center, University of South Alabama Mitchell Cancer Institute, Mobile, AL 36604, USA; xichen@health.southalabama.edu (X.C.); ymaxuitenko@health.southalabama.edu (Y.Y.M.); akeeton@health.southalabama.edu (A.B.K.); gpiazza@health.southalabama.edu (G.A.P.); 2 ADT Pharmaceuticals, Orange Beach, AL 36561, USA

**Keywords:** drug targets/oncoprotein and tumor suppressor drug targets, drug mechanisms, cell signaling/guanine nucleotide binding proteins and effectors, oncogenes and tumor suppressors/KRAS

## Abstract

Oncogenic mutations in *RAS* genes result in the elevation of cellular active RAS protein levels and increased signal propagation through downstream pathways that drive tumor cell proliferation and survival. These gain-of-function mutations drive over 30% of all human cancers, presenting promising therapeutic potential for RAS inhibitors. However, many have deemed RAS “undruggable” after nearly 40 years of failed drug discovery campaigns aimed at identifying a RAS inhibitor with clinical activity. Here we review RAS nucleotide cycling and the opportunities that RAS biochemistry presents for developing novel RAS inhibitory compounds. Additionally, compounds that have been identified to inhibit RAS by exploiting various aspects of RAS biology and biochemistry will be covered. Our current understanding of the biochemical properties of RAS, along with reports of direct-binding inhibitors, both provide insight on viable strategies for the discovery of novel clinical candidates with RAS inhibitory activity.

## 1. Introduction

*RAS* mutations are among the most common mutations in cancer, driving aggressive, metastatic malignancies with poor patient prognoses. Notably, pancreatic (95%), colorectal (45%), and lung (35%) cancers harbor *KRAS* mutations at remarkably high frequencies. Mutations in *RAS* genes are also known to cause developmental disorders of the heart and nervous system, known as RASopathies [1]. RAS remains an elusive drug target despite its well-characterized role in cancer and extensive efforts to develop novel therapeutics targeting RAS-driven cancers. Multiple aspects of RAS structural biology present challenges for the development of small molecule inhibitors, including a lack of deep, druggable pockets, an ultra-high affinity for its guanine nucleotide substrates, and few structural differences between wild-type and oncogenic RAS proteins [1]. Attempts to target RAS directly or by its post-translational modifications and association with the plasma membrane have either failed in the development process or have not been fully characterized [2]. Oncogenic RAS is present predominantly in its active guanosine triphosphate (GTP)-bound state, due to impaired GTP hydrolysis activity. The elevation of RAS-GTP levels in *RAS* mutant tumors causes increased activation of its vast array of downstream effectors, promoting cell signal transduction pathways, and facilitating proliferation and survival [3].

A number of anti-cancer drugs that block a multitude of signaling nodes, either upstream or downstream of RAS, have been developed and approved for clinical use by the United States Food and Drug Administration (FDA). However, these therapies have limited clinical utility for RAS-driven cancers, and often result in the reoccurrence of highly aggressive cancers that are resistant to chemotherapy or radiation [4]. Inhibitors that directly target RAS and inhibit its ability to activate complex downstream signaling pathways are expected to have strong efficacy and safety advantages over currently available upstream or downstream inhibitors of RAS signaling.

## 2. The *RAS* Gene Family

The *RAS* proto-oncogene family (*KRAS*, *NRAS*, and *HRAS*) encodes RAS proteins, which are critical components of cell signal transduction pathways that regulate proliferation and survival. Mutations in *RAS* genes form the active *RAS* oncogenes, which are found in 30% of human cancers. The discovery of transforming viruses in the 1960s, which potently induced rat sarcomas, provided the first clues of the existence of these oncogenes that are now known to drive a number of aggressive human cancers [5,6]. The name *RAS* was later given to this oncogene family due to its ability to promote rat sarcoma formation. The names of the *HRAS* and *KRAS* genes were derived from those responsible for their discoveries, Harvey, and Kirsten, respectively. Meanwhile the *NRAS* gene was assigned its name after its discovery in DNA isolated from a neuro-fibroma cell line [7].

Activating missense mutations in *KRAS* account for 85% of all mutations among the three *RAS* genes, while *NRAS* mutations represent 12%, and *HRAS* mutations represent 3%. Mutations of each isoform are exclusive of each other in tumor cells, and the individual isoform that is mutated in a particular tumor cell has been shown to exhibit a strong preference to its tissue of origin. For example, *RAS* mutations in pancreatic cancer are almost exclusively *KRAS* mutations (greater than 95%), *NRAS* mutations are the predominant *RAS* mutations in melanoma (94%), and *HRAS* mutations are the most common *RAS* mutations in bladder cancers (54%) [7,8]. In addition to the bias of individual isoform mutations to specific tumor types, the three isoforms can also be distinguished by their most commonly mutated codon. For example, 80% of *KRAS* mutations are codon 12 mutations, meanwhile 60% of *NRAS* mutations occur at codon 61. *HRAS* mutations have less bias toward a specific codon with 50% occurring at codon 12, and 40% found at codon 61 [9]. Some specific *RAS* mutations show high prevalence in particular tumor types, with the *KRAS* G12D mutation found in 44% of colorectal cancers and 39% of pancreatic cancers, while 59% of non-small cell lung cancers harbor *KRAS* G12C mutations [8]. This prevalence of specific isoform and codon mutations presents opportunities for the development of RAS inhibitors with high selectivity for tumor cells harboring a particular *RAS* mutation. The discovery of selective *KRAS* G12C inhibitors presents great promise for the treatment of lung cancers that are driven by this mutation, but these inhibitors will not be effective for other cancers with lower prevalence of *KRAS* G12C mutations, such as colorectal (12%) and pancreatic (4%) cancers [10].

KRAS, NRAS, and HRAS proteins all contain highly conserved N-terminal GTPase domains or G-domains that are identical through their first 86 amino acids [2]. This first portion of the G-domain, also known as the effector lobe, contains the active site for GTPase hydrolysis activity, along with two switch regions that are essential for regulator and effector binding. The most significant conformational changes associated with nucleotide cycling occur in these switch regions, with switch I consisting of amino acids 30-40 and switch II containing amino acids 60-68. Sequence divergence between isoforms resides in the last 80 amino acids of the G-domain, referred to as the allosteric lobe, and the C-terminal hypervariable region located within amino acids 167-188. Additionally, KRAS proteins exist as two alternative splicing variants, KRAS4A and KRAS4B. KRAS4B is the predominantly expressed variant in human tumors, and is therefore, more intensely studied [11,12].

The allosteric lobe and the C-terminal hypervariable region have both been shown to be involved in isoform-specific RAS-membrane interactions that have critical roles in propagating RAS signaling [13]. The C-terminal region of RAS proteins ends with a CAAX motif, with the C representing a cysteine, A representing any aliphatic amino acid, and X representing any amino acid. The cysteine is isoprenylated by farnesyltransferases or geranylgeranyl transferases as a form of post-translational processing [14,15]. These isoprenoid chains are critical for RAS association with the plasma membrane. The addition of the farnesyl group is the primary form of isoprenylation. However, in the absence of farnesyl transferase activity, KRAS and NRAS, but not HRAS, can be geranylgeranylated. After isoprenylation, an endopeptidase cleaves the AAX residues from the C-terminus, and the previously isoprenylated C-terminal cysteine is also methylated by a methyltransferase [16]. After these initial steps of post-translational processing, RAS moves to the plasma membrane, where a palmitoyltransferase catalyzes the addition of two palmitoyl fatty acid chains to a cysteine just upstream of the C-terminal cysteine that was previously farnesylated and methylated. These palmitoyl fatty acid chains then associate with the hydrophobic inner portion of the plasma membrane. KRAS4B is not palmitoylated, but instead relies on several lysine residues in close proximity to its C-terminus (polybasic region) to interact with the negative charges on plasma membrane phospholipid head groups [3].

## 3. RAS Nucleotide Cycling

**Figure 1:**
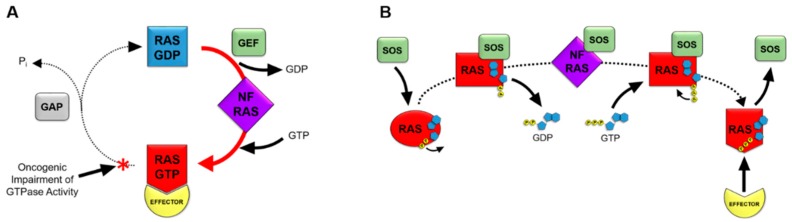
RAS as a molecular switch in the cell. Figure 1 depicts the mechanisms of RAS nucleotide cycling. RAS proteins cycle from an inactive GDP-bound state to a nucleotide-free (NF) transition state, followed by formation of the active GTP-bound state (**A**). Guanine nucleotide exchange factors (GEFs) facilitate the exchange of GDP for GTP. The conformation of RAS is dynamic through this exchange, with GTP-bound RAS being in an active conformation that binds effectors containing RAS binding domains. The intrinsic GTPase activity of RAS hydrolyzes GTP to GDP in order to revert back to the inactive state of RAS. GTPase activating proteins (GAPs) promote GTP hydrolysis activity by approximately 1,000-fold. Oncogenic mutations in *RAS* genes impair the GTPase activity of RAS, resulting in a prevalence of the active, GTP-bound state of RAS in tumor cells. This portion of the cycle prevalent in oncogenic RAS is depicted with a red arrow and asterisk. SOS mediates the exchange of GDP for GTP on RAS proteins (**B**). SOS binding to RAS results in a conformational shift in RAS, occluding the magnesium cofactor from interacting with the phosphate groups of GDP. The resulting decrease in affinity of RAS for GDP contributes to the release of GDP to form the nucleotide-free (NF) state of RAS. GTP binding to RAS subsequently occurs due to high concentrations of GTP in tumor cells. Initially, only the guanosine and ribose moieties of GTP bind to RAS. Interactions of the gamma phosphate group of GTP with the magnesium cofactor of RAS result in displacement of SOS. The resulting active conformation of GTP-bound RAS can interact with effectors containing RAS binding domains to activate downstream signaling pathways.

RAS proteins are molecular switches that cycle between an inactive, guanosine diphosphate (GDP)-bound state and an active, guanosine triphosphate (GTP)-bound state (Figure 1A). RAS binds guanine nucleotides with ultra-high affinity and relies on interactions with regulatory proteins that catalyze transitions between inactive and active states under physiological conditions. Throughout the process of nucleotide cycling, RAS undergoes a number of dynamic conformational changes that offer new opportunities for inhibition with small molecules.

Guanine nucleotide exchange factors, or GEFs, associate with RAS-GDP and decrease the affinity of RAS for GDP when RAS is in an inactive, GDP-bound state. The GEF that is typically associated with RAS nucleotide exchange is known as Son of Sevenless, or SOS. The catalytic site of SOS inserts an α-helix into the switch 1 domain of RAS, resulting in an opening of the nucleotide-binding pocket of RAS. This α-helix of SOS contains side chains that also interact with the switch 2 region of RAS, displacing residues within the active site of RAS. The resulting conformational shift partially occludes the magnesium cofactor of RAS from the active site, and therefore, disrupts the hydrophilic interactions between the magnesium ion and the phosphate moieties of the GDP substrate. GDP then dissociates from RAS to produce a RAS nucleotide-free state. Nucleotide-free RAS has previously believed to be a transient state of RAS, but it has been shown to be a physiologically relevant participant in cell signal transduction pathways [17]. The nucleotide-free state of RAS is a unique, unstable conformation. The presence of SOS complexed with nucleotide-free RAS is known to increase its stability [18]. The conformation of RAS as part of the nucleotide-free RAS-SOS complex has been shown to closely mimic the conformation of a GDP-bound RAS protein that is missing its magnesium cofactor, causing the loss of coordination between the magnesium ion and the phosphate groups of the GDP substrate as a result of SOS binding [19].While, the RAS-SOS complex occludes phosphate groups of guanine nucleotides from interacting with the magnesium cofactor, it does not block the ribose or guanine moieties of guanine nucleotide substrates from binding to RAS. This manner in which RAS-SOS binding does not completely block the active site allows the ribose and guanine portions of the incoming GTP substrate to bind RAS (Figure 1B).

High intracellular levels of GTP result in GTP binding to RAS while in a nucleotide-free conformation. Subsequently, interactions formed between the gamma phosphate of GTP and the magnesium cofactor of RAS result in a conformational change of RAS that dissociates SOS from the complex [20]. Once GTP is bound, the conformation of RAS has been reported to be in a dynamic state between two conformations. The first of these conformations being an “open and off” conformation, known as state 1. This conformation consists of a more open active site with loose GTP binding. Meanwhile, the second “closed and on” conformation, or state 2, consists of tighter GTP binding that is considered to be the truly active conformation for signal propagation through effector binding [21]. Two highly conserved switch I residues, tyrosine-32 and threonine-35, have been reported to coordinate the state of GTP binding, while also participating in initiation of the GTPase reaction that hydrolyzes GTP back to GDP. Tight interaction of these residues with the phosphates of GTP, along with threonine-35 coordinating the position of the magnesium cofactor in the active site, has been reported to produce the state 2 conformation of GTP-bound RAS [22,23,24,25,26]. When RAS is in the state 2 conformation, effector proteins with RAS binding domains (RBDs) bind to switch I and switch II in the effector lobe of the G-domain. RAS-effector binding then initiates downstream signaling cascades, such as the MAPK or PI3K/AKT pathways. The activated conformational change, induced by GTP-binding, is reversible by means of the RAS-mediated hydrolysis of GTP to GDP [27]. The intrinsic GTPase activity of RAS is slow, but can be accelerated more than 1000-fold by the association of RAS with GAPs, or GTPase activating proteins. GAPs contain a residue referred to as the arginine finger that is known to catalyze the GTPase reaction by inserting into the active site of RAS [28]. This arginine residue is conserved among all of the RAS GAPs. The arginine finger associates with glycine-12 and glutamine-61 of RAS by van der Waals interactions. The positively charged side chain of the arginine steers it towards the negatively charged phosphate groups of GTP, even though it does not shift the charge distribution of the GTP molecule prior to catalysis [29]. The interaction of the GAP, with the switch II region of RAS, positions glutamine-61 to participate with glycine-12 and the GAP arginine finger in the catalytic reaction [30]. These interactions between RAS and the GAP arginine finger push water molecules out of the active site, resulting in a charge shift in the GTP molecule that aids in the cleavage of the gamma phosphate [31]. The arginine finger participates in the cleavage reaction itself by neutralizing the charges formed as part of the reaction mechanism, and therefore, stabilizing the transition state of the cleavage reaction [30]. The GAP dissociates from RAS after the release of the gamma phosphate, suggesting that its presence is critical not just for the cleavage of the terminal phosphate bond, but also the subsequent release of the gamma phosphate [31].

The kinetics of nucleotide cycling in both wild-type and mutant RAS proteins have been well-characterized. Notably, GTP loading of nucleotide-free RAS occurs at a similar rate in both wild-type and oncogenic RAS. In the case of oncogenic RAS, a single missense mutation in the active site impairs the ability of RAS to hydrolyze GTP. The mutations typically occur in glycine-12 or glutamine-61 of RAS, with mutations in either residue causing disruptions of substrate interactions with RAS and the GAP arginine finger that are critical for enzymatic activity. As a result of the mutation, the altered conformation of the active site of RAS inhibits the ability of the GAP to insert its arginine finger into the RAS active site. Although, mutant RAS is often referred to as “constitutively active,” these proteins retain the ability to revert to an inactive, GDP-bound state. Their GAP-stimulated GTPase activity is reduced by 97–99% in comparison to wild-type RAS. Collectively, this impairment of GTPase activity results in the prevalence of the active, GTP-bound RAS state. Therefore, oncogenic activation produces increased RAS-effector binding and stimulation of downstream signaling pathways [32]. The current understanding of the complex dynamics of RAS nucleotide cycling presents prospective opportunities for exploiting RAS structural biology and biochemistry for the development of small molecule inhibitors.

## 4. The RAS Signal Transduction Pathway

**Figure 2:**
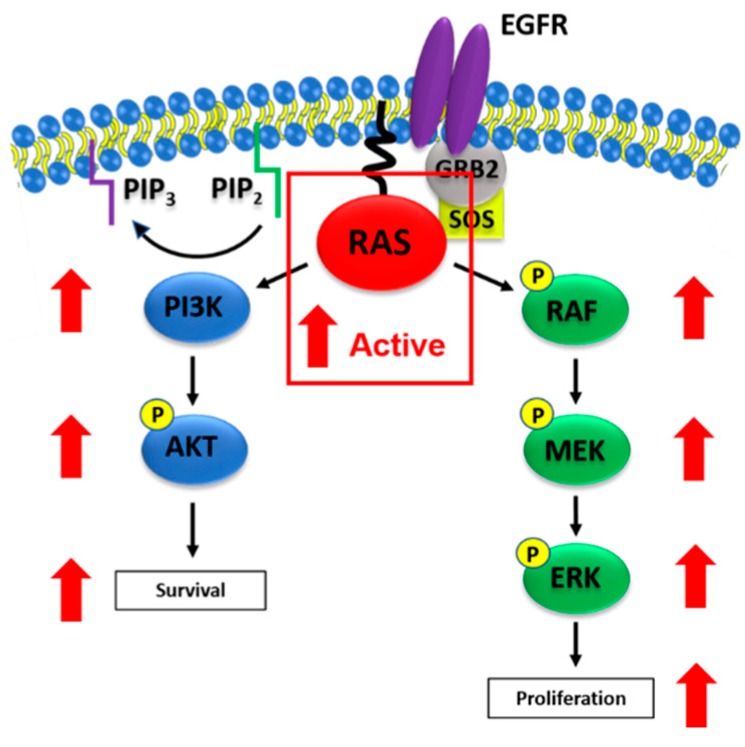
Oncogenic RAS activation of MAPK and PI3K/AKT signaling. Figure 2 illustrates oncogenic RAS-mediated activation of MAPK and PI3K/AKT signaling. Mutant RAS proteins are prevalent in their active, GTP-bound states in tumor cells, resulting in elevated activation of downstream signaling cascades. Active RAS interacts with the RAS binding domain (RBD) of RAF to activate MAPK signaling, which is typically associated with tumor cell proliferation. RAS can also interact with the RBD of PI3K in order to activate PI3K/AKT signaling and promote tumor cell survival. Red arrows indicate elevations of phosphorylated protein levels in the presence of mutant RAS (outlined in the red box).

In a cellular context, RAS activation occurs when a ligand binds to the extracellular region of a receptor tyrosine kinase (RTK), such as the epidermal growth factor receptor (EGFR), resulting in autophosphorylation of intracellular tyrosine residues. The adaptor protein growth factor receptor-bound protein 2 (GRB2) contains a SH2 domain that binds phosphorylated tyrosine residues in the intracellular domain of EGFR. Additionally, SH3 domains within GRB2 bind to the RAS GEF, SOS. The localization of SOS to the plasma membrane places it in close proximity with RAS, which is also localized to the membrane as a result of post-translational processing. SOS subsequently catalyzes RAS nucleotide exchange, facilitating the replacement of GDP with GTP and shifting its structure to the active state 2 GTP-bound conformation. This active conformation is the predominant state of oncogenic RAS in *RAS* mutant tumors [3]. At this point, active RAS interacts with and activates effectors containing RBDs, such as RAF serine/threonine protein kinases and type I phosphatidylinositol 3-kinases (PI3Ks), with high affinity binding [33,34]. RAF and PI3K subsequently initiate signaling cascades through the MAPK and PI3K/AKT signaling pathways, respectively. The well-defined MAPK pathway is known to increase expression of cell cycle regulatory proteins to drive cell cycle progression. Meanwhile, PI3K/AKT signaling plays a key role in anti-apoptotic signaling and promotes cell survival [7]. Mutant RAS stimulates these signaling pathways in the absence or presence of RTK stimulation (Figure 2). MAPK and PI3K/AKT signaling both contain nodes that participate in cross-talk between the two pathways. This cross-talk limits the therapeutic potential of inhibitors targeting individual pathway components by creating mechanisms of compensation that are often difficult to interpret [35]. The challenges in inhibiting individual pathways suggest that targeting RAS is a promising treatment option by blocking multiple signaling pathways that are essential for malignant progression. In cases where RAS inhibitors target specific mutations, such as covalent KRAS G12C inhibitors, combinations with inhibitors of other pathway components may be necessary for efficacy. For example, resistance to KRAS G12C inhibitors in pre-clinical models of lung cancer has been shown to be overcome by combined PI3K inhibition [36]. Additionally, it is worth noting that *RAS* mutant tumor cells also express the wild-type versions of the other two RAS isoforms. For example, a *KRAS* mutant tumor cell still expresses wild-type NRAS and HRAS proteins. These wild-type isoforms have been shown to drive RAS signaling independently of oncogenic RAS under certain conditions of growth factor stimulation [37]. This suggests that in the case of mutation-specific RAS inhibitors, such as KRAS G12C inhibitors, RAS inhibitors that target the other wild-type isoforms may be necessary to completely block RAS signaling.

## 5. Approaches to Inhibit RAS

The discovery of direct-binding RAS inhibitors has been a priority of oncology research in both academia and the pharmaceutical industry for more than four decades. Multiple biochemical and structural characteristics of RAS have proved to be difficult hurdles for inhibitors to overcome, leading many to describe RAS as an “undruggable” target. RAS lacks deep, druggable pockets that would provide opportunities for small molecule binding. Picomolar affinities for binding guanine nucleotides, along with millimolar concentrations of GTP in tumor cells, present challenges for developing competitive inhibitors that bind within the catalytic domain. Also, oncogenic RAS has very minute structural differences from wild-type RAS, possessing only a single codon missense mutation in the G-domain. However, this specific nature of *RAS* mutations introduces opportunities to develop drugs that are potentially selective for tumor cells harboring mutant RAS [13]. A drug with RAS-dependent tumor cell selectivity would have attractive therapeutic benefits such as the possibility for strong antitumor efficacy, low potential for toxicity, and the potential to inhibit the growth of drug-resistant tumors with activated RAS.

Indirectly targeting RAS by inhibiting biological processes associated with its subcellular localization or its upstream and downstream signal propagation once seemed to be promising prospects for drug discovery. Attempts to target post-translational processing and localization to the plasma membrane, which are both essential for RAS function, with farnesyl transferase inhibitors were early approaches at targeting RAS. Farnesyl transferase inhibitors were ineffective in phase II and III clinical trials for the treatment of pancreatic cancer due to alternative KRAS prenylation by geranylgeranyl transferases [1,38]. Dual inhibition of farnesyl transferase and geranylgeranyl transferase was not able to be achieved below dose-limiting toxicity in animal models of pancreatic cancer [39,40]. Targeting upstream receptor tyrosine kinases such as EGFR has been a success for targeted therapy in tumor types such as lung and colorectal cancers. However, tumors with activating downstream *RAS* mutations are resistant to EGFR inhibition, leading clinicians to use the *RAS* mutation status of patients’ tumors as a predictor of a negative response to anti-EGFR therapies [41]. FDA-approved inhibitors of upstream receptor tyrosine kinases can even induce *RAS* mutations as a mechanism of tumor cell chemoresistance [42]. Targeting downstream components of RAS signaling with inhibitors of MAPK signaling or inhibitors of the PI3K/AKT/mTOR pathway has been another approach to block RAS signaling that can produce an initial clinical response. However, this response is rarely long-lasting, due to compensatory feedback mechanisms resulting in drug-resistant, highly aggressive tumors. While some inhibitors of downstream signaling such as MEK inhibitors show promising activity in select pre-clinical models of RAS-driven cancer, dose-limiting toxicities reduce their ability to show clinical efficacy as monotherapies [43]. 

**Figure 3:**
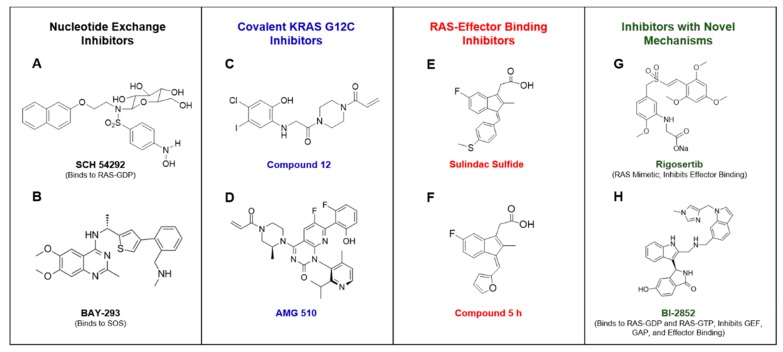
Portrays compounds that have been reported to inhibit RAS or RAS signaling by various mechanisms. SCH 54292 was reported to bind GDP-bound RAS and inhibit nucleotide exchange [41] (**A**). BAY-293 was found to directly bind to SOS and inhibit RAS nucleotide exchange [45] (**B**). Compound 12 was the first covalent KRAS G12C selective inhibitor to be identified [50] (**C**). AMG 510 is a covalent KRAS G12C inhibitor that is currently in clinical trials [55] (**D**). Sulindac sulfide has been reported to weakly bind to RAS and inhibit RAS-effector binding [67] (**E**). Compound 5 h is a sulindac derivative that was found to have improved RAS binding activity in comparison to the parent compound [74] (**F**). Rigosertib was described as a RAS mimetic that inhibits RAS-mediated activation of effectors [64] (**G**). BI-2852 was reported to bind to the switch I/II pocket of RAS-GDP and RAS-GTP to inhibit GEF, GAP, and effector binding to RAS [75] (**H**).

Direct-acting RAS inhibitors may circumvent the limitations of previous attempts to drug *RAS* mutant tumors, but no such inhibitors have received FDA approval. Nonetheless, numerous strategies have been explored in attempt to exploit RAS biochemical properties and associated biological functions to directly inhibit its oncogenic activity. Compounds that block the exchange of GDP for GTP have been discovered by several groups. A series of compounds that bind GDP-bound RAS to inhibit nucleotide exchange were the first to be reported to directly bind to RAS, with the lead compound being SCH 54292 (Figure 3A). These compounds were found to bind a previously undescribed cleft in the switch II region of GDP-bound RAS [44]. Others have employed fragment-based discovery strategies to identify molecules that inhibit SOS-mediated nucleotide exchange. These fragments take advantage of either the SOS binding site on RAS, or binding sites created by formation of the RAS-SOS complex [45,46,47]. Bayer AG recently underwent a discovery campaign aimed at targeting SOS directly to inhibit SOS-mediated RAS nucleotide exchange. The lead compound that was discovered, BAY-293, inhibited RAS-SOS binding with IC_50_ values in the low nanomolar range (Figure 3B). While, this compound is a first-in-class potent inhibitor of SOS-RAS interactions, its lack of *in vivo* bioavailability limit its development potential [48]. However, these findings demonstrate that compounds that directly bind to SOS can inhibit RAS activation, and therefore, RAS signaling, with high potency. SOS-mediated nucleotide exchange in response to receptor tyrosine kinase stimulation has been shown to be inhibited by SHP2 inhibitors. SHP2 is a regulator of SOS that has been characterized to contribute to the resistance to MEK inhibition in *RAS* mutant mouse tumor models. This data is supported by the observation of synergy with combined MEK and SHP2 inhibition in *in vivo* models of pancreatic and lung cancer [49,50]. A series of small molecules reported to directly inhibit GTP binding to RAS and other GTPases was discovered using a bead-based flow cytometry screen. The lead compound, CID1067700, shows activity in cell-free biochemical assays with IC_50_ values in the nanomolar range. The compound lacks potency in cells but has the potential to serve as a molecular probe for use in further studies of RAS inhibition in cell-free models [51]. Another group identified a peptide that selectively inhibits SOS-mediated nucleotide exchange of KRAS G12D by screening with a custom phage display peptide library [52]. While currently known inhibitors of nucleotide exchange show promise, their pitfalls include low binding affinities and lack of activity in cell-based assays [2]. Nucleotide exchange inhibitors that bind to RAS with high affinity and retain activity in RAS-mutant cells are presumably be candidates for clinical development.

Recent drug discovery campaigns aiming for mutant RAS-selective inhibitors represent perhaps the most substantial progress towards an FDA-approved RAS inhibitor. A fragment-based screening approach identified a novel pocket adjacent to the active site in the KRAS G12C mutant protein. This allosteric site is susceptible to covalent binding of a novel inhibitor, compound 12 (Figure 3C), which depends on the mutant cysteine residue for binding. The resulting covalent interaction displaces the switch I and switch II regions of RAS and causes preferential binding of GDP, therefore, locking RAS in an inactive state [10]. Additional reports have described compounds that use similar strategies to selectively target the KRAS G12C mutant, providing the opportunity for a high therapeutic index [53,54]. Others have used a common approach of covalently targeting the G12C cysteine, but instead using GDP analogs with electrophilic groups present on the β phosphate [55,56]. Two novel covalent inhibitors that specifically target KRAS G12C, Amgen’s AMG 510 and Mirati Therapeutics’ MRTX849, are currently in phase I clinical trials. In a presentation at the 2019 American Society of Clinical Oncology (ASCO) meeting, AMG 510 was reported to produce partial responses in 50% of patients with *KRAS* G12C mutant non-small cell lung cancer, while also contributing to stable disease in approximately 77% of enrolled patients with *KRAS* G12C mutant colorectal or appendix cancers (Figure 3D) [57].

Targeting the nucleotide-free state of RAS is a relatively unexplored strategy for inhibiting nucleotide loading of RAS. This state of RAS only seems to exist transiently in cells prior to GTP loading. However, a recent report has shown that nucleotide-free RAS exists in a large enough fraction in cells to participate in signal transduction, as it can indeed bind to and negatively regulate the activity of class II PI3Ks. This interaction in turn negatively regulates RAS activation by blocking GTP binding to RAS and locking RAS in a nucleotide-free conformation [17]. The negative regulation of RAS activation by effectors binding and trapping RAS in an inactive, nucleotide-free state suggests that similar approaches can be taken using pharmacological inhibition. In fact, a synthetic α-helix, HBS3, was identified to inhibit GTP loading by binding to nucleotide-free RAS in the portion of the switch regions where SOS binding occurs [58]. Additionally, BIM-46187 has been reported as a novel compound with cell permeability that locks heterotrimeric G-proteins in a nucleotide-free state [59]. Nucleotide-free RAS has been reported to be unstable and subject to spontaneous denaturation [18,60]. These thermodynamic properties present challenges for the identification of inhibitors selective for nucleotide-free RAS. However, targeting this state of RAS presents attractive opportunities in tumor cells by freezing RAS in an unstable conformation and potentially promoting a reduction of RAS protein levels. Taken together, nucleotide-free RAS represents an underappreciated, physiologically relevant state of RAS that shows evidence of being druggable by novel inhibitors.

Another promising approach for directly targeting RAS is disrupting the interaction between active, GTP-bound RAS and its effectors [61]. Yeast two-hybrid screens were utilized by two independent groups to identify a class of small molecule inhibitors, along with an antibody fragment, that inhibits RAS-effector binding [62,63]. A cyclic peptide known as cyclorasin has also been identified and shown to competitively block effector binding to RAS [64]. Another group employed in silico docking screens to identify a compound, 3144, that inhibits RAS-RAF interactions with impressive in vitro and in vivo activity in *KRAS* mutant tumor models [65]. However, drug-like properties, such as potency, solubility, and molecular weight limitations will need to be improved for further development of this compound. Rigosertib, a polo-like kinase inhibitor, was discovered to unexpectedly act as a RAS mimetic to inhibit binding of RAS to RBDs of a multitude of effectors, including RAF kinases and PI3K (Figure 3G). However, rigosertib lacks selective inhibition of RAS-induced transformation, as it additionally inhibits transformation induced by various subunits of PI3K independently of RAS activation [66]. Currently, rigosertib is in various phases of clinical trials for patients with select hematologic cancers. Another group identified a class of “miniproteins” that bind RAS with nanomolar affinity to inhibit effector binding. These inhibitory proteins cannot cross cell membranes, but could serve as tools for the biochemical characterization of novel inhibitors of RAS-effector binding [67]. Additionally, two novel compounds known as PPIN-1 and PPIN-2 were recently identified to inhibit RAS-effector interactions by a screen that employed compound soaking with KRAS crystals. These compounds were synthesized by combining moieties of previously identified inhibitors of RAS-effector interactions with fragments of an anti-RAS antibody. They were found to target a hydrophobic pocket near the switch I domain of RAS and show improved potency in vitro and in cell-based assays in comparison to their parent molecules [68].

The cyclooxygenase inhibitor, sulindac sulfide (Figure 3E), has been shown to inhibit RAS-RAF binding [69]. Although, the binding affinity is low and requires high micromolar concentrations, such concentrations are comparable to those required to inhibit tumor cell growth [70,71]. Sulindac sulfone, the non-cyclooxygenase (COX) inhibitory metabolite of sulindac, also exhibits anti-cancer activity in mutant *RAS* cell-based assays, which suggest that the mechanism is unrelated to COX inhibition [72,73]. While, there is no in vitro evidence that sulindac sulfide is RAS selective, its parent compound sulindac and sulindac sulfone have been reported to selectively inhibit the formation of *RAS* mutant tumors in a chemical-induced rat model of breast cancer [74]. Attempts have been made to modify the sulindac scaffold to improve inhibition of RAS-effector binding. These efforts have produced sulindac derivatives with improved inhibition of RAS-RAF binding and reduced COX inhibition. The lead compound, compound 5 h (Figure 3F), was confirmed by NMR to bind to a region of RAS that is critical for RAS-RAF binding [75,76]. These sulindac derivatives have low potency, exhibiting micromolar IC_50_ values for cell growth inhibition and inhibition of RAS-RAF binding.Additionally, they may have other binding partners that contribute to their anticancer activity at micromolar concentrations. However, these studies suggest that chemical modification of sulindac to further improve RAS inhibitory activity and eliminate COX inhibitory activity represents a promising approach to target RAS for anti-tumor activity with reduced potential for toxicities.

While most discovery strategies target one specific aspect of RAS nucleotide cycling, a novel inhibitor was recently discovered that inhibits several protein-protein interactions that are critical for RAS functionality. This compound binds to a shallow pocket between the switch I and switch II domains of RAS that was previously thought to be “undruggable.” The novel compound, BI-2852, binds to both the RAS-GDP and RAS-GTP states to inhibit GEFs, GAPs, and effectors all from interacting with RAS [77]. This demonstrated ability to block RAS from cycling, regardless of whether it is GDP or GTP-bound, presents a promising opportunity to develop more potent inhibitors with high potential for efficacy in RAS-driven tumors.

## 6. Conclusions

Despite extensive efforts to identify small molecule inhibitors of RAS, there are currently no FDA-approved drugs that directly bind to RAS and selectively inhibit RAS-driven tumor growth. The cycling of guanine nucleotides to shift RAS between inactive and active conformations provides new opportunities to target RAS. The most successful approaches, thus far, have been covalently trapping RAS in its inactive GDP-bound state, along with inhibiting RAS-effector binding. Selective covalent inhibitors of the KRAS G12C mutant, such as AMG 510, elicited promising results in clinical trials for the treatment of lung and colorectal cancers. Additionally, the RAS mimetic rigosertib is currently being evaluated in clinical trials for the treatment of hematologic cancers. While the progress towards developing clinically effective RAS inhibitors is promising, the therapeutic potential of compounds targeting specific mutants is limited to subsets of RAS-driven cancers. The current understanding of the mechanism of RAS nucleotide exchange presents numerous opportunities for reversible inhibitors, with several recent discovery campaigns producing novel reversible RAS inhibitors. A reversible RAS inhibitor would be highly significant to the oncology field at large and extremely valuable for the treatment of patients harboring *RAS* mutations other than G12C. Taken together, significant progress has been made towards drugging the “undruggable” RAS, and the hunt continues for a promising clinical candidate that could be the first FDA-approved RAS inhibitor.

## References

[1] Prior I.A., Lewis P.D., Mattos C. (2012). A comprehensive survey of Ras mutations in cancer. Cancer Res..

[2] Stephen A.G., Esposito D., Bagni R.K., McCormick F. (2014). Dragging Ras Back in the Ring. Cancer Cell.

[3] Holderfield M. (2017). Efforts to Develop KRAS Inhibitors. Cold Spring Harb. Perspect. Med..

[4] Downward J. (2003). Targeting RAS signalling pathways in cancer therapy. Nat. Rev. Cancer.

[5] Nash G.M., Gimbel M., Shia J., Nathanson D.R., Ndubuisi M.I., Zeng Z.S., Kemeny N., Patye P.B. (2010). Kras Mutation Correlates with Accelerated Metastatic Progression in Patients with Colorectal Liver Metastases. Ann. Surg. Oncol..

[6] Harvey J.J. (1964). An Unidentified Virus which causes the Rapid Production of Tumours in Mice. Nature.

[7] Kirsten W.H., Mayer L.A. (1967). Morphologic Responses to a Murine Erythroblastosis Virus. J. Natl. Cancer Inst..

[8] Cox A.D., Der C.J. (2010). Ras History: The Saga Continues. Small GTPases.

[9] Cox A.D., Fesik S.W., Kimmelman A.C., Luo J., Der C.J. (2014). Drugging the undruggable RAS: Mission Possible?. Nat. Rev. Drug Discov..

[10] Ostrem J.M., Peters U., Sos M.L., Wells J.A., Shokat K.M. (2013). K-Ras(G12C) inhibitors allosterically control GTP affinity and effector interactions. Nature.

[11] Buhrman G., O′connor C., Zerbe B., Kearney B.M., Napoleon R., Kovrigina E.A., Vajda S., Kozakov D., Kovrigin E.L., Mattos C. (2011). Analysis of Binding Site Hot Spots on the Surface of Ras GTPase. J. Mol. Biol..

[12] Hobbs G.A., Der C.J., Rossman K.L. (2016). RAS isoforms and mutations in cancer at a glance. J. Cell Sci..

[13] Parker J.A., Mattos C. (2015). The Ras–Membrane Interface: Isoform-Specific Differences in the Catalytic Domain. Mol. Cancer Res..

[14] Casey P.J., Solski P.A., Der C.J., Buss J.E. (1989). p21ras is modified by a farnesyl isoprenoid. Proc. Natl. Acad. Sci. USA.

[15] Farnsworth C.C., Gelb M.H., Glomset J.A. (1990). Identification of Geranylgeranyl-Modified Proteins in HeLa Cells. Science.

[16] Gutierrez L., Magee A.I., Marshall C.J., Hancock J.F. (1989). Post-translational processing of p21ras is two-step and involves carboxyl-methylation and carboxy-terminal proteolysis. EMBO J..

[17] Wong K.A., Russo A., Wang X., Chen Y.J., Lavie A., O’Bryan J.P. (2012). A New Dimension to Ras Function: A Novel Role for Nucleotide-Free Ras in Class II Phosphatidylinositol 3-Kinase Beta (PI3KC2β) Regulation. PLoS ONE.

[18] John J., Sohmen R., Feuerstein J., Linke R., Wittinghofer A., Goody R.S. (1990). Kinetics of interaction of nucleotides with nucleotide-free H-ras p21. Biochemistry.

[19] Mori K., Hata M., Neya S., Hoshino T. (2005). Common Semiopen Conformations of Mg^2+^-Free Ras, Rho, Rab, Arf, and Ran Proteins Combined with GDP and Their Similarity with GEF-Bound Forms. J. Am. Chem. Soc..

[20] Boriack-Sjodin P.A., Margarit S.M., Bar-Sagi D., Kuriyan J. (1998). The structural basis of the activation of Ras by Sos. Nature.

[21] Ye M., Shima F., Muraoka S., Liao J., Okamoto H., Yamamoto M., Tamura A., Yagi N., Ueki T., Kataoka T. (2005). Crystal Structure of M-Ras Reveals a GTP-Bound “Off” State Conformation of Ras Family Small GTPases. J. Biol. Chem..

[22] Valencia A., Chardin P., Wittinghofer A., Sander C. (1991). The ras protein family: Evolutionary tree and role of conserved amino acids. Biochemistry.

[23] Geyer M., Schweins T., Herrmann C., Prisner T., Wittinghofer A., Kalbitzer H.R. (1996). Conformational Transitions in p21Ras and in Its Complexes with the Effector Protein Raf-RBD and the GTPase Activating Protein GAP. Biochemistry.

[24] Spoerner M., Herrmann C., Vetter I.R., Kalbitzer H.R., Wittinghofer A. (2001). Dynamic properties of the Ras switch I region and its importance for binding to effectors. Proc. Natl. Acad. Sci. USA.

[25] Shima F., Ijiri Y., Muraoka S., Liao J., Ye M., Araki M., Matsumoto K., Yamamoto N., Sugimoto T., Yoshikawa Y. (2010). Structural Basis for Conformational Dynamics of GTP-bound Ras Protein. J. Biol. Chem..

[26] Matsumoto S., Miyano N., Baba S., Liao J., Kawamura T., Tsuda C., Takeda A., Yamamoto M., Kumasaka T., Kataoka T. (2016). Molecular Mechanism for Conformational Dynamics of Ras·GTP Elucidated from In-Situ Structural Transition in Crystal. Sci. Rep..

[27] Cherfils J., Zeghouf M. (2013). Regulation of Small GTPases by GEFs, GAPs, and GDIs. Physiol. Rev..

[28] Ahmadian M.R., Stege P., Scheffzek K., Wittinghofer A. (1997). Confirmation of the arginine-finger hypothesis for the GAP-stimulated GTP-hydrolysis reaction of Ras. Nat. Genet..

[29] Heesen H.T., Gerwert K., Schlitter J. (2007). Role of the arginine finger in Ras·RasGAP revealed by QM/MM calculations. FEBS Lett..

[30] Scheffzek K. (1997). The Ras-RasGAP Complex: Structural Basis for GTPase Activation and Its Loss in Oncogenic Ras Mutants. Science.

[31] Kötting C., Kallenbach A., Suveyzdis Y., Wittinghofer A., Gerwert K. (2008). The GAP arginine finger movement into the catalytic site of Ras increases the activation entropy. Proc. Natl. Acad. Sci. USA.

[32] Hunter J.C., Manandhar A., Carrasco M.A., Gurbani D., Gondi S., Westover K.D. (2015). Biochemical and Structural Analysis of Common Cancer-Associated KRAS Mutations. Mol. Cancer Res..

[33] Herrmann C., Martin G.A., Wittinghofer A. (1995). Quantitative Analysis of the Complex between p21 and the Ras-binding Domain of the Human Raf-1 Protein Kinase. J. Biol. Chem..

[34] Rodriguez-Viciana P., Warne P.H., Dhand R., Vanhaesebroeck B., Gout I., Fry M.J., Waterfield M.D., Downward J. (1994). Phosphatidyl-3-OH Kinase as a Direct Target of Ras. Nature.

[35] Mendoza M.C., Er E.E., Blenis J. (2011). The Ras-ERK and PI3K-mTOR pathways: Cross-talk and compensation. Trends Biochem. Sci..

[36] Misale S., Fatherree J.P., Cortez E., Li C., Bilton S.J., Timonina D., Myers D.T., Lee D., Gomez-Caraballo M., Greenberg M. (2018). KRAS G12C NSCLC Models Are Sensitive to Direct Targeting of KRAS in Combination with PI3K Inhibition. Clin. Cancer Res..

[37] Young A., Lou D., McCormick F. (2013). Oncogenic and Wild-type Ras Play Divergent Roles in the Regulation of Mitogen-Activated Protein Kinase Signaling. Cancer Discov..

[38] Baines A.T., Xu D., Der C.J. (2011). Inhibition of Ras for cancer treatment: The search continues. Future Med. Chem..

[39] Lobell R.B., Liu N., Buser C.A., Davide J.P., DePuy E., Hamilton K., Koblan K.S., Lee Y., Mosser S., Motzel S.L. (2002). Preclinical and clinical pharmacodynamic assessment of L-778,123, a dual inhibitor of farnesyl:protein transferase and geranylgeranyl:protein transferase type-I. Mol. Cancer Ther..

[40] Desolms S.J., Ciccarone T.M., MacTough S.C., Shaw A.W., Buser C.A., Ellis-Hutchings M., Fernandes C., Hamilton K.A., Huber H.E., Kohl N.E. (2003). Dual Protein Farnesyltransferase−Geranylgeranyltransferase-I Inhibitors as Potential Cancer Chemotherapeutic Agents. J. Med. Chem..

[41] Raponi M., Winkler H., Dracopoli N.C. (2008). KRAS mutations predict response to EGFR inhibitors. Curr. Opin. Pharmacol..

[42] Knickelbein K., Zhang L. (2015). Mutant KRAS as a critical determinant of the therapeutic response of colorectal cancer. Genes Dis..

[43] Gysin S., Salt M., Young A., McCormick F. (2011). Therapeutic Strategies for Targeting Ras Proteins. Genes Cancer.

[44] Taveras A., Remiszewski S., Doll R., Cesarz D., Huang E., Kirschmeier P., Pramanik B., Snow M., Wang Y.S., Del Rosario J. (1997). Ras oncoprotein inhibitors: The discovery of potent, ras nucleotide exchange inhibitors and the structural determination of a drug-protein complex. Bioorg. Med. Chem..

[45] Maurer T., Garrenton L.S., Oh A., Pitts K., Anderson D.J., Skelton N.J., Fauber B.P., Pan B., Malek S., Stokoe D. (2012). Small-molecule ligands bind to a distinct pocket in Ras and inhibit SOS-mediated nucleotide exchange activity. Proc. Natl. Acad. Sci. USA.

[46] Sun Q., Burke J.P., Phan J., Burns M.C., Olejniczak E.T., Waterson A.G., Lee T., Rossanese O.W., Fesik S.W. (2012). Discovery of Small Molecules that Bind to K-Ras and Inhibit Sos-Mediated Activation. Angew. Chem..

[47] Winter J.J.G., Anderson M., Blades K., Brassington C., Breeze A.L., Chresta C., Embrey K., Fairley G., Faulder P., Finlay M.R.V. (2015). Small Molecule Binding Sites on the Ras:SOS Complex Can Be Exploited for Inhibition of Ras Activation. J. Med. Chem..

[48] Hillig R.C., Sautier B., Schroeder J., Moosmayer D., Hilpmann A., Stegmann C.M., Werbeck N.D., Briem H., Boemer U., Weiske J. (2019). Discovery of potent SOS1 inhibitors that block RAS activation via disruption of the RAS–SOS1 interaction. Proc. Natl. Acad. Sci. USA.

[49] Ruess D.A., Heynen G.J., Ciecielski K.J., Ai J., Berninger A., Kabacaoglu D., Görgülü K., Dantes Z., Wörmann S.M., Diakopoulos K.N. (2018). Po-201 mutant kras-driven cancers depend on ptpn11/shp2 phosphatase. Nat. Med..

[50] Lu H., Liu C., Velazquez R., Wang H., Dunkl L.M., Kazic-Legueux M., Haberkorn A., Billy E., Manchado E., Brachmann S.M. (2019). SHP2 Inhibition Overcomes RTK-Mediated Pathway Re-Activation in KRAS Mutant Tumors Treated with MEK Inhibitors. Mol. Cancer Ther..

[51] Hong L., Guo Y., Basuray S., Agola J.O., Romero E., Simpson D.S., Schroeder C.E., Simons P., Waller A., Garcia M. (2015). A Pan-GTPase Inhibitor as a Molecular Probe. PLoS ONE.

[52] Sakamoto K., Kamada Y., Sameshima T., Yaguchi M., Niida A., Sasaki S., Miwa M., Ohkubo S., Sakamoto J.I., Kamaura M. (2017). K-Ras(G12D)-selective inhibitory peptides generated by random peptide T7 phage display technology. Biochem. Biophys. Res. Commun..

[53] Lito P., Solomon M., Hansen R., Li L.S., Rosen N. (2016). Abstract LB-071: Allele-specific inhibitors inactivate mutant KRAS G12C by a trapping mechanism. Science.

[54] Patricelli M.P., Janes M.R., Li L.S., Hansen R., Peters U., Kessler L.V., Chen Y., Kucharski J.M., Feng J., Ely T. (2016). Selective Inhibition of Oncogenic KRAS Output with Small Molecules Targeting the Inactive State. Cancer Discov..

[55] Lim S.M., Westover K.D., Ficarro S.B., Harrison R.A., Choi H.G., Pacold M.E., Carrasco M., Hunter J., Kim N.D., Xie T. (2014). Therapeutic Targeting of Oncogenic K-Ras by a Covalent Catalytic Site Inhibitor. Angew. Chem. Int. Ed. Engl..

[56] Xiong Y., Lu J., Hunter J., Li L., Scott D., Choi H.G., Lim S.M., Manandhar A., Gondi S., Sim T. (2017). Covalent Guanosine Mimetic Inhibitors of G12C KRAS. ACS Med. Chem. Lett..

[57] (2019). Research AA for C. AMG 510 First to Inhibit “Undruggable” KRAS. https://cancerdiscovery.aacrjournals.org/content/early/2019/06/11/2159-8290.CD-NB2019-073.

[58] O’Bryan J.P. (2019). Pharmacological Targeting of RAS: Recent Success with Direct Inhibitors. Pharmacol. Res..

[59] Schmitz A.L., Schrage R., Gaffal E., Charpentier T.H., Wiest J., Hiltensperger G., Morschel J., Hennen S., Häußler D., Horn V. (2014). A cell-permeable inhibitor to trap Gαq proteins in the empty pocket conformation. Chem. Biol..

[60] Müller M.P., Jeganathan S., Heidrich A., Campos J., Goody R.S. (2017). Nucleotide based covalent inhibitors of KRas can only be efficient in vivo if they bind reversibly with GTP-like affinity. Sci. Rep..

[61] Keeton A.B., Salter E.A., Piazza G.A. (2017). The RAS-Effector Interaction as a Drug Target. Cancer Res..

[62] Kato-Stankiewicz J., Hakimi I., Zhi G., Zhang J., Serebriiskii I., Guo L., Edamatsu H., Koide H., Menon S., Eckl R. (2002). Inhibitors of Ras/Raf-1 interaction identified by two-hybrid screening revert Ras-dependent transformation phenotypes in human cancer cells. Proc. Natl. Acad. Sci. USA.

[63] Tanaka T., Rabbitts T.H. (2010). Interfering with RAS–effector protein interactions prevent RAS-dependent tumour initiation and causes stop–start control of cancer growth. Oncogene.

[64] Trinh T.B., Upadhyaya P., Qian Z., Pei D. (2016). Discovery of a Direct Ras Inhibitor by Screening a Combinatorial Library of Cell-Permeable Bicyclic Peptides. ACS Comb. Sci..

[65] Welsch M.E., Kaplan A., Chambers J.M., Stokes M.E., Bos P.H., Zask A., Zhang Y., Sanchez-Martin M., Badgley M.A., Huang C.S. (2017). Multivalent Small-Molecule Pan-RAS Inhibitors. Cell.

[66] Athuluri-Divakar S.K., Carpio R.V.D., Dutta K., Baker S.J., Cosenza S.C., Basu I., Gupta Y.K., Reddy M.R., Ueno L., Hart J.R. (2016). A Small Molecule RAS-Mimetic Disrupts RAS Association with Effector Proteins to Block Signaling. Cell.

[67] McGee J.H., Shim S.Y., Lee S.J., Swanson P.K., Jiang S.Y., Durney M.A., Verdine G.L. (2018). Exceptionally High-Affinity Ras Binders That Remodel Its Effector Domain. J. Biol. Chem..

[68] Cruz-Migoni A., Canning P., Quevedo C.E., Bataille C.J.R., Bery N., Miller A., Russell A.J., Phillips S.E.V., Carr S.B., Rabbitts T.H. (2019). Structure-based development of new RAS-effector inhibitors from a combination of active and inactive RAS-binding compounds. Proc. Natl. Acad. Sci. USA.

[69] Herrmann C., Block C., Geisen C., Haas K., Weber C., Winde G., Möröy T., Müller O. (1998). Sulindac sulfide inhibits Ras signaling. Oncogene.

[70] Piazza G.A., Rahm A.L., Krutzsch M., Sperl G., Paranka N.S., Gross P.H., Brendel K., Burt R.W., Alberts D.S., Pamukcu R. (1995). Antineoplastic drugs sulindac sulfide and sulfone inhibit cell growth by inducing apoptosis. Cancer Res..

[71] Piazza G.A., Rahm A.K., Finn T.S., Fryer B.H., Li H., Stoumen A.L., Pamukcu R., Ahnen D.J. (1997). Apoptosis primarily accounts for the growth-inhibitory properties of sulindac metabolites and involves a mechanism that is independent of cyclooxygenase inhibition, cell cycle arrest, and p53 induction. Cancer Res..

[72] Lawson K.R., Ignatenko N.A., Piazza G.A., Cui H., Gerner E.W. (2000). Influence of K-ras activation on the survival responses of Caco-2 cells to the chemopreventive agents sulindac and difluoromethylornithine. Cancer Epidemiol. Biomark. Prev..

[73] Piazza G.A., Keeton A.B., Tinsley H.N., Gary B.D., Whitt J.D., Mathew B., Thaiparambil J., Coward L., Gorman G., Li Y. (2009). A novel sulindac derivative that does not inhibit cyclooxygenases but potently inhibits colon tumor cell growth and induces apoptosis with antitumor activity. Cancer Prev. Res..

[74] Thompson H.J., Jiang C., Lu J., Mehta R.G., Piazza G.A., Paranka N.S., Pamukcu R., Ahnen D.J. (1997). Sulfone metabolite of sulindac inhibits mammary carcinogenesis. Cancer Res..

[75] Karaguni I.M., Glüsenkamp K.H., Langerak A., Geisen C., Ullrich V., Winde G., Möröy T., Müller O. (2002). New indene-derivatives with anti-proliferative properties. Bioorg. Med. Chem. Lett..

[76] Waldmann H., Karaguni I.M., Carpintero M., Gourzoulidou E., Herrmann C., Brockmann C., Oschkinat H., Müller O. (2004). Sulindac-Derived Ras Pathway Inhibitors Target the Ras–Raf Interaction and Downstream Effectors in the Ras Pathway. Angew. Chem. Int. Ed..

[77] Kessler D., Gmachl M., Mantoulidis A., Martin L.J., Zoephel A., Mayer M., Gollner A., Covini D., Fischer S., Gerstberger T. (2019). Drugging an undruggable pocket on KRAS. Proc. Natl. Acad. Sci. USA.

